# Expanding the Secondary Use of Prostate Cancer Real World Data: Automated Classifiers for Clinical and Pathological Stage

**DOI:** 10.3389/fdgth.2022.793316

**Published:** 2022-06-02

**Authors:** Selen Bozkurt, Christopher J. Magnani, Martin G. Seneviratne, James D. Brooks, Tina Hernandez-Boussard

**Affiliations:** ^1^Department of Medicine (Biomedical Informatics), Stanford University, Stanford, CA, United States; ^2^School of Medicine, Stanford University, Stanford, CA, United States; ^3^Department of Biomedical Data Sciences, Stanford University, Stanford, CA, United States

**Keywords:** prostate cancer, TNM stage, natural language processing, machine learning, stage

## Abstract

**Background:**

Explicit documentation of stage is an endorsed quality metric by the National Quality Forum. Clinical and pathological cancer staging is inconsistently recorded within clinical narratives but can be derived from text in the Electronic Health Record (EHR). To address this need, we developed a Natural Language Processing (NLP) solution for extraction of clinical and pathological TNM stages from the clinical notes in prostate cancer patients.

**Methods:**

Data for patients diagnosed with prostate cancer between 2010 and 2018 were collected from a tertiary care academic healthcare system's EHR records in the United States. This system is linked to the California Cancer Registry, and contains data on diagnosis, histology, cancer stage, treatment and outcomes. A randomly selected sample of patients were manually annotated for stage to establish the ground truth for training and validating the NLP methods. For each patient, a vector representation of clinical text (written in English) was used to train a machine learning model alongside a rule-based model and compared with the ground truth.

**Results:**

A total of 5,461 prostate cancer patients were identified in the clinical data warehouse and over 30% were missing stage information. Thirty-three to thirty-six percent of patients were missing a clinical stage and the models accurately imputed the stage in 21–32% of cases. Twenty-one percent had a missing pathological stage and using NLP 71% of missing T stages and 56% of missing N stages were imputed. For both clinical and pathological T and N stages, the rule-based NLP approach out-performed the ML approach with a minimum F1 score of 0.71 and 0.40, respectively. For clinical M stage the ML approach out-performed the rule-based model with a minimum F1 score of 0.79 and 0.88, respectively.

**Conclusions:**

We developed an NLP pipeline to successfully extract clinical and pathological staging information from clinical narratives. Our results can serve as a proof of concept for using NLP to augment clinical and pathological stage reporting in cancer registries and EHRs to enhance the secondary use of these data.

## Introduction

Prostate cancer is the most common solid-organ malignancy in men, with over 160,000 new cases expected in the United States in 2020 (1). Cancer care for these men can be complicated, costly, and fragmented. Patients often need to navigate across multiple providers, settings of care, and levels of complex treatment regimens and cancer stage is critical in guiding prognosis and treatment options. Explicit documentation of cancer stage within a patient's health record is a quality metric endorsed by the National Quality Forum and the Quality Oncology Practice Initiative (QOPI) by the American Society for Clinical Oncology (ASCO) (2, 3).

While critical to support evidence-based patient care, patients' medical records and cancer registries are often missing or have inaccurate staging information (4–6). Stage information is missing from 10 to 50% of patient records in cancer registries likely because of absent documentation of explicit stage in patients' medical records (7–9). However, the data used to derive cancer stage is often recorded in unstructured text within the electronic health records (EHR). While the unstructured data provides clinicians opportunities to elaborate on the patient's clinical and/or pathological stage, information found within the unstructured text make it less accessible for secondary use (10–12). Furthermore, when stage is documented only as unstructured text, it requires labor-intensive manual abstraction by trained registrars to obtain the information from patient medical records, which is both costly and prone to error (13–15). In addition, the requirement for manual review results in significant delays between the point of care and registry updates.

Missing stage information substantially constrains the secondary use of these real-world data sources since these cases must be excluded from any analysis, threatening generalizability. The availability of stage across a greater percentage of registry patients could improve capture of population-level distributions across an entire EHR population and allow data synchronization across different institutions and data ecosystems. Automated stage extraction could also reduce costs associated with manual extraction currently used to populate local, state and national registries. Automated stage extraction from EHRs could benefit patient care by sharing accurate diagnostic data across treating institutions or by improving performance of clinical decision-support tools designed to recommend evidence-based treatments. The 21st Century Cures Act encourage incorporating real-world data sources, such as that extracted from the EHR, into clinical assertions (10, 16, 17). Therefore, there is an urgent need for the adoption of advanced informatics methodologies such as machine learning (ML) and natural language processing (NLP) to unlock the information embedded within free clinical text of the EHR.

Few previous works have addressed prostate cancer stage extraction from clinical text (11, 12). An important limitation of stage extraction models developed to date is their focus on specific TNM staging patterns such as “pT1N2M0.” In particular, such models only consider single occurrences of these patterns and do not learn from the context around those specific expressions. In the real-world, descriptions of stage information may be complicated (see below). For such examples, simple pattern matching based feature selection would not be sufficient as the information needed for staging is embedded into free text.

**Table:** 

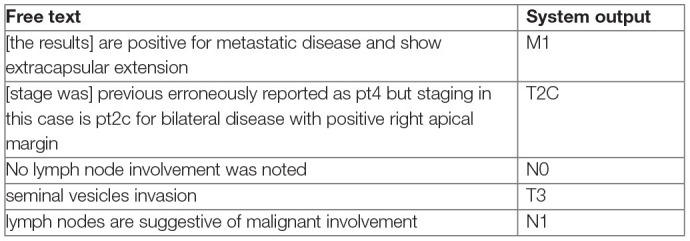

In this study, we develop and evaluate an NLP framework to extract clinical and pathological stage from the free-text clinical narratives of prostate cancer patients at a tertiary academic medical center. We then tested whether this approach could augment stage information within a regional cancer registry. Our approach could serve as a framework for wider use of NLP for real-world data and guide strategies for automated stage extraction from unstructured clinical text.

## Methods

An overall schema* of the study design is illustrated in Supplementary Material 1.

### Dataset

Data were collected from a prostate cancer Clinical Data Warehouse (CDW), which is described in detail elsewhere (18). In brief, data were collected from a tertiary care academic healthcare system's EHR records (Epic Systems, Verona, Wisconsin, USA) that were linked to the California Cancer Registry, which contains data on diagnosis, histology, cancer stage, treatment and outcomes. The stage information in our CDW was collected from three sources: (1) the hospital cancer registry, (2) structured staging fields in the EHR system, (3) the California Cancer Registry (CCR). Stage was assigned as “missing” if it was not present in any of these sources.

### Cohort Selection

**Figure 1:**
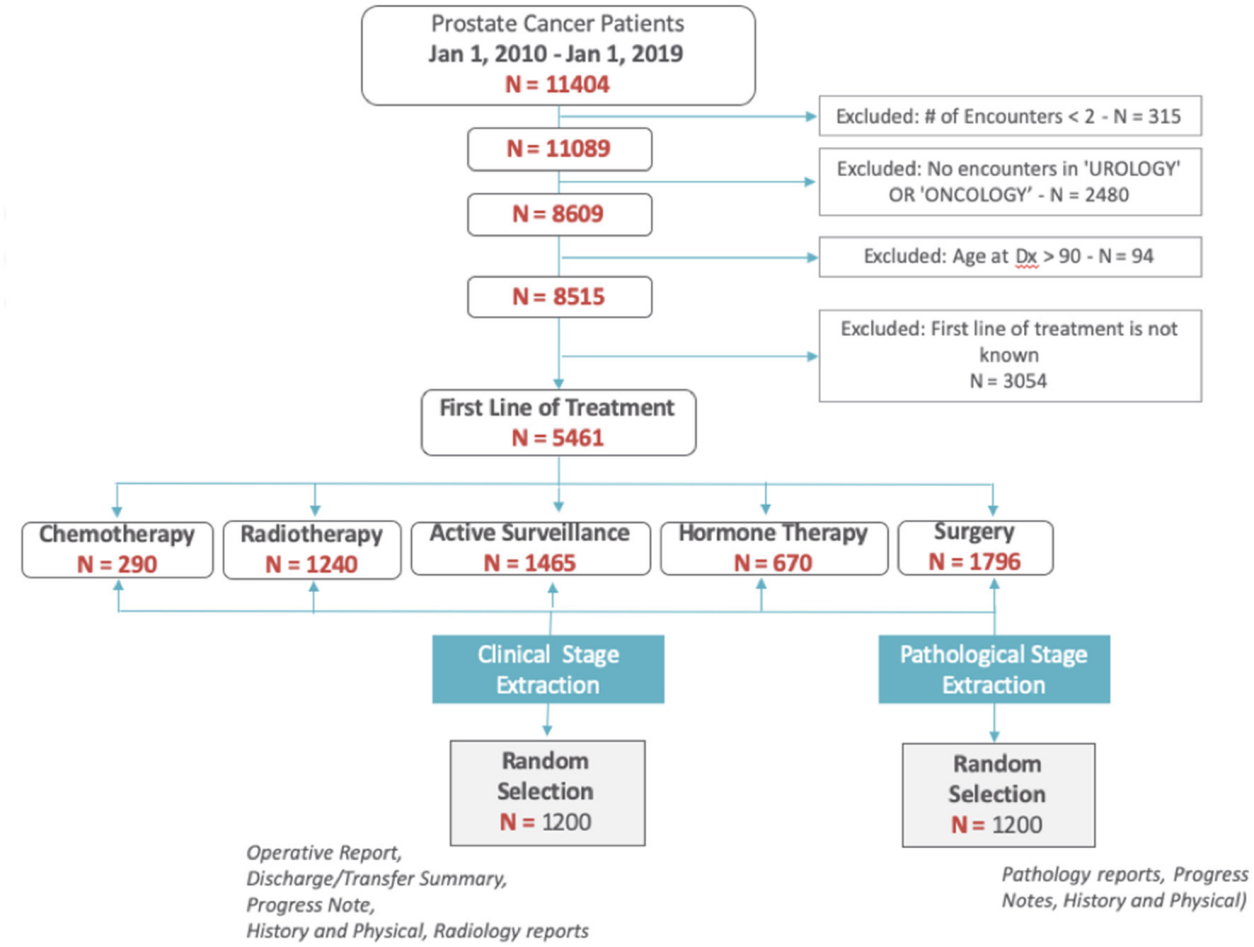
Cohort selection diagram.

We identified patients diagnosed with prostate cancer between 2010 and 2018. We excluded patients with less than two encounters recorded in the EHR, without visits to urology or oncology clinics, those missing a recorded first line of treatment, or who were older than 90-years (Figure 1).

### Manual Annotations

Among the 5,461 patients with prostate cancer, we randomly selected 2,400 patients (1,200 for clinical staging; 1,200 for pathological staging) to establish a set with known staging status (ground truth) to be used for training and validating the NLP methods. Staging information was abstracted from patients' clinical narratives, pathology and radiology reports *via* chart review by both a trained nurse and clinical fellow. Only o*perative reports, history and physical notes, discharge/transfer summaries*, and *progress notes* were used to abstract clinical stage. For pathological stage, only *pathology reports, history and physical notes*, and *progress notes* were used. Patient-level agreement between annotators across was calculated using Cohen's kappa agreement score. We also calculated the agreement between manual annotations by our annotators and the data collected from our cancer registry.

### Main Outcomes

Clinical and pathological stage was assigned for the primary tumor in the prostate, whether there were lymph node, and distant metastasis (TNM) in accord with the American Joint Committee on Cancer (AJCC, 7th edition) recommendations, the most widely used cancer staging system (17). There were seven classification labels used across clinical and pathological staging. The distribution of T stage categories were substantially unbalanced therefore each stage was dichotomized into a binary classification task: clinical T stage (1–2 and 3–4), clinical N stage (0 and 1), clinical M stage (0 and 1), pathological T stage (2 and 3–4) and pathological N stage (0 and 1).

### Natural Language Processing Pipeline

**Figure 2:**
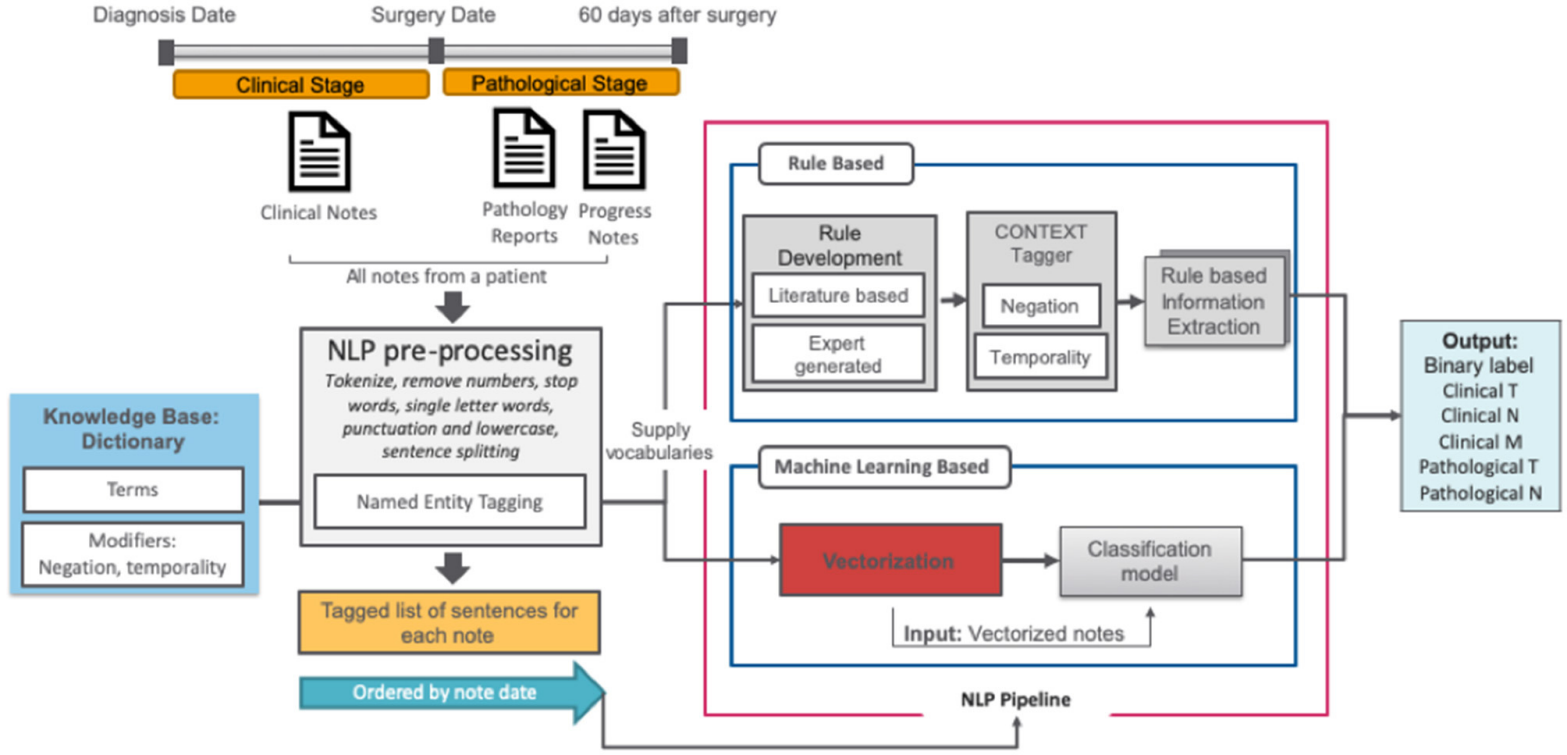
NLP pipelines.

The NLP pipeline consisted of a set of subtasks outlined in Figure 2 and is described in further detail below. For the classification task, two alternate approaches were compared: (1) rule-based and (2) semi-supervised machine learning.

### Knowledge Base

In order to capture a broad vocabulary used for cancer staging, we extended the TNM terminology with two complementary dictionaries: (1) the target term list (*n* = 156), curated by clinical experts and additional terms primarily captured through a semi-supervised trained dictionary analysis of clinical notes in the CDW that we describe elsewhere (19), and (2) the modifier list, a publicly available set of modifier terms that includes terms related to negations, temporality, and discussion (20).

### Pre-processing

All clinical notes were pre-processed using basic text cleaning steps, implemented using the NLTK library. Pre-processing was initiated with sentence boundary detection and tokenization, then all punctuation characters and words <2 letters were removed. Integer and floating-point numbers were converted to a corresponding string representation. After pre-processing, all reports for a given patient were ordered by date and concatenated.

### Rule-Based Approaches

Firstly, all related clinical terms were identified using staging guidelines from the American Joint Committee on Cancer (AJCC, 7th edition) and our expert urologists' recommendations. These clinical terms were used as the target terms by the rule-based algorithm. Those target terms are often modified by several contextual properties relevant to our information extraction task; ConText (20) identifies three contextual values—hypothetical, historical, and experiencer—in addition to negation *via* NegEx. We implemented ConText within the NLP system to determine whether a stage entity is negated and its temporal status.

### Machine Learning Models

We used the keywords-based document-level vector representations of the text to train a classifier against the T, N, and M stage labels from the manually annotated set. The pre-processed clinical notes from the training set were used to create vector embeddings for words in a completely unsupervised manner using the word2vec model (21). For word2vec training, we used the skip-gram model with vector length 100 and window width 5, and default settings for all other parameters as we reported related experiments in our previous paper (22). We then searched for keywords in each report and, if a match was found, we defined its context as the sentence where the term was found (23). The context's vector was then computed by averaging its constituent word vectors using the pretrained word2vec embeddings. Using the vector representation of text for each patient, we used support vector machines (SVM) as a binary classifier. We used random hyperparameter search to find optimal inputs to the classifiers with F1-score as the target metric.

### Model Evaluation

We compared NLP pipeline results with the manual chart review values using the 1,200 patients random sample, collecting true positives (TP), true negatives (TN), false positives (FP), and false negatives (FN). Pipeline performance was evaluated in terms of precision, recall, and F1-score. An 80/20% split was used between training/test sets, and 5-fold cross validation was applied.

### Clinical Utility Evaluation

To evaluate pipeline utility, we targeted missing staging information in the remaining 4,261 patients whose records were not included in the manually annotated gold standard set. Using these records as an input, we evaluated the results of the NLP pipeline in terms of percentage of missing stages imputed.

## Results

### Patient Characteristics

There were 5,461 prostate cancer patients in our cohort who received first line therapies at our center (Table 1). Median age at diagnosis was 67 (35–90). The majority (70%) of patients were non-Hispanic white, and more than half (58%) were insured by Medicare.

**Table 1:** Cohort characteristics (*n* = 5,461).

**Characteristics**			**Median (Min-Max)/n (%)**
Age			67 (35–90)
Race/ethnicity	White		3,846 (70)
	Asian		612 (11)
	Hispanic/Latino		460 (8)
	Black		261 (5)
	Other		282 (5)
Insurance type	Private		1,818 (33)
	Medicare		3,148 (58)
	Medicaid		181 (3)
	Other		314 (6)
Clinical stage	T	1	2,405 (44)
		2	1,059 (19)
		3	160 (3)
		4	40 (1)
		Missing	1,797 (33)
	N	0	3,321 (61)
		1	148 (3)
		Missing	1,992 (36)
	M	0	3,409 (62)
		1	275 (5)
		Missing	1,777 (33)
Pathological stage (only for	T	2	909 (51)
surgery patients *n* = 1,796)		3–4	539 (30)
		Missing	348 (19)
	N	0	1,353 (75)
		1	51 (3)
		Missing	392 (22)

Based on data fields that were populated in the CDW, clinical stage was predominantly T1 (44%), N0 (61%) and M0 (62%). Similarly, the most common pathological stage was T2 (51%) and N0 (75%). Since pathological stage is assigned only for patients with localized prostate cancer who undergo radical prostatectomy, fewer than ten patients had a pathological M stage of 1, so these were excluded from the analysis. For clinical staging, 33% of T, 30% of N and 33% of M staging fields were missing in the EHR. For pathological staging, 19% of T and 22% of N staging information was missing from the EHR. For the period of 2010–2019, the highest proportion (42%) of cases with missing staging information were in 2018, the most recent eligible year.

### Model Evaluation Results

Inter-rater agreement (kappa coefficient) between the two reviewers for the manual chart review of 1,200 patients was 0.85 for clinical staging and 0.95 for pathological staging.

The rule-based model outperformed the ML model for clinical T and N staging with F1-scores over 0.71 (see Table 2). However, ML models achieved better results for clinical M stage than the rule-based model, with an F1-score of 0.98 for M0 and 0.88 for M1. For pathological T stage classification, both models achieved similar results with F1-scores over 0.85 except for classification of N1 stage, as the ML model failed to correctly classify N1 cases while the rule-based model reached F1-score of 0.88.

**Table 2:** Evaluation of NLP models.

**NLP Approach**	**Stages**	**Categories**	**Clinical Stage**			**Pathological Stage**		
			**Precision**	**Recall**	**F1**	**Precision**	**Recall**	**F1**
Rule based model	T	T1-T2	0.98	0.95	0.97	0.95	0.94	0.94
		T3-T4	0.74	0.88	0.80	0.90	0.91	0.90
	N	N0	0.97	0.99	0.98	0.99	0.94	0.97
		N1	0.91	0.59	0.71	0.85	0.92	0.88
	M	M0	0.97	0.98	0.97	–	–	–
		M1	0.81	0.76	0.79	–	–	–
Machine Learning Model	T	T1-T2	0.95	0.69	0.80	0.89	0.96	0.92
		T3-T4	0.27	0.75	0.40	0.92	0.80	0.85
	N	N0	0.98	0.80	0.88	0.96	0.97	0.97
		N1	0.26	0.82	0.40	0.20	0.17	0.18
	M	M0	0.98	0.98	0.98	–	–	–
		M1	0.86	0.89	0.88	–	–	–

### Augmentation of Clinical and Pathological Stage in the Clinical Data Warehouse (CDW)

We compared clinical and pathological stage between the ground truth (manual chart review) and the CDW, *N* = 1,200. Clinical T stage showed low agreement (Kappa Score 0.64) and pathological T and N stages showed excellent agreement (Kappa score 0.98). For clinical stage, a main cause of disagreement was the documentation of pathological stage instead of clinical stage when there is a pathological stage available for the same patient. Another source of disagreement was ambiguous documentation of staging such as “he has t3a prostate cancer” or “stage 1 prostate cancer,” as it is unclear if this is clinical or pathological stage. We further evaluated the agreement of the NLP model with structured cancer registry data in the CDW for the remaining cases not used for model training and testing. Agreement for clinical T, N and M stages were 0.64, 0.78, 0.86, respectively and agreement for pathological T and N stages were 0.84, 0.83, respectively. Agreement for clinical N and M stages was not calculated due to the small number of cases in both data sources.

To further quantify the performance of the NLP models, we evaluated the top performing model's ability to impute missing CDW stage information (Table 3). The CDW is missing stage information for over 20% of patients. The NLP model imputed clinical T, N, and M stage category in 24, 21, and 32% of the missing records, respectively. For pathological staging, the NLP model imputed 71% of missing T stages and 56% of missing N stages. In total, the NLP model extracted 30% (1,882/6,306) of missing clinical and pathological stages in the CDW from clinical notes.

**Table 3:** Missing data imputation statistics.

**Stage**	**Total *N***	**Missing *N* (%)**	**Stage categories**	**Imputed**	**Imputation (%)**
Clinical T	5,461	1,797 (33)	1–2	309	24
			3–4	119	
Clinical N	5,461	1,992 (36)	0	303	21
			1	124	
Clinical M	5,461	1,777 (33)	0	470	32
			1	91	
Pathological T	1,796	348 (19)	2	100	71
			3–4	148	
Pathological N	1,796	392 (22)	0	155	56
			1	63	

## Discussion

Cancer stage is a critical piece of information underpinning prognosis and treatment decisions for cancer patients, yet it is often not readily available within real-world data. Using a clinical data warehouse at a comprehensive cancer that linked EHRs with cancer registry data, we found that the discrete documentation of clinical and pathological stage was missing for over one-third of prostate cancer patients. This level of missing data significantly impairs clinical work flow and the secondary use of these real-world data sources, motivating the development of an NLP pipeline to identify and extract both clinical and pathological stages from clinical narratives in the EHR. The NLP models achieved excellent performance for both clinical and pathological stage information, with rule-based methods consistently outperforming machine learning models. Furthermore, the pipeline was able to augment staging documentation missing in the CDW. This approach can be applied to any healthcare system's prostate cancer patient population to enhance staging documentation and secondary EHR use.

With incentives provided under the Affordable Care Act, the secondary use of EHRs has increased dramatically (24). EHRs were developed for billing purposes and are designed to act as central repositories of structured data such as laboratory values and house unstructured data such as physician notes. EHRs also provide opportunities for secondary uses including identification of patients for clinical trial enrollment, conducting pragmatic clinical trials, carrying out post-market surveillance, monitoring and improving adherence to clinical guidelines, cost analyses and population-based studies (19, 22, 25, 26). Furthermore, emerging evidence suggests that clinical care could be improved through EHR-based automated decision-making aides and risk calculators to facilitate personalized care at the bedside (27). However, despite these opportunities some of the most essential metrics to define patient care, such as cancer staging, is often not easily accessible in the EHRs as a discrete field (18, 19). Rather, clinical and pathological staging information is distributed across diverse types of clinical notes, such as the physical examination, treatment plans, or pathology and radiology reports, requiring NLP solutions to accommodate diverse data sources. Beyond decision support, accurate staging data are essential for clinical trial recruitment and population-wide studies. Hence, robust NLP methods are needed to collect complete and accurate stage information.

The models we develop are unique in extracting both clinical and pathological stage, allowing the capture of missing staging data from the entire spectrum of prostate cancer patients to enable robust secondary analyses. Pathological stage is better recorded in the EHRs and often available with smart text phrases in the unstructured data, providing opportunities for NLP approached. Hence, most of the recent studies, used NLP models specifically for prostate cancer stage extraction from clinical narratives, have exclusively focused on extracting pathological stage (12, 28–33). Since pathological stage is limited to those patients undergoing surgical removal of the prostate (radical prostatectomy), these studies fail to capture staging information for prostate cancer patients receiving alternative treatments, such as radiation therapy, hormonal therapy, chemotherapy or active surveillance. However, clinical and pathological stage provide separate information necessary for clinicians to classify prostate cancer patients (34). In this study, both clinical and pathological stages information were extracted form clinical narratives, however, the NLP models were less accurate in assigning clinical T and N stages compared to pathological stages. Clinical staging can be difficult to accurately capture manually, since it involves parsing physical examination features such as findings on prostate physical examination and interpretation of subtle findings on imaging studies (34). This challenge was highlighted by the relatively low agreement (*k* = 0.64) in clinical T stage assignment between the records manually labeled by clinical experts and the cancer registry. Common reasons for mis-assignment of stage in the cancer registry included assignment of pathological stage to the clinical stage field or ambiguous stage information in the clinical notes such as assignment of different clinical stages across the clinical notes.

To improve the impact and utility of clinical NLP tasks, it is important that applications are developed at the patient-level, which can be challenging because this requires the synthesis of information in clinical narrative text from the sentence-level to the document-level to the patient-level. Another common limitation of previous staging algorithms is that they usually identify stage at the sub-document (e.g., sentence) or document level (35). In contrast to previous work, the NLP methods in this study extract stage at the patient level. While this is more meaningful for clinical care and population studies, it requires the model to successfully distinguish longitudinal information from competing reports. Using a rule-based and semi-supervised ML approach, we achieved promising results that build upon and expand previous studies. To the authors' knowledge, this study is the first to report both clinical and pathological stage at the patient level using all the clinical notes from the first diagnosis to treatment.

In this study, the rule-based classifier showed superior performance metrics for clinical and pathological T and N stages compare to the ML approach. However, for clinical M stage extraction, we demonstrate superior performance using a semi-supervised ML approach, in contrast to recent studies which predominantly use rule-based approaches for all types of stage extraction (12, 28–33). ML methods can have several advantages over rule-based approaches in terms of generalizability and may be successfully applied across different stage types (clinical and pathological), that are typically derived from different kinds of reports where the structure of text varies. While a rule-based approach may require a domain-specific dictionary which can be site-specific, a ML approach applies learning techniques that are not specific to a practice or healthcare setting. The performance of ML models may be limited by variability in the textual descriptions of tumor size and lymph node metastases, which is the key factor determining T and N stages, respectively. Extracting T and N stages proved to be more challenging with ML model, and further work using larger and more diverse training and test sets is warranted.

The use of sophisticated machine learning (ML) tools and techniques utilizing artificial intelligence with the enormous amount of data available in the modern EHR provides new opportunities to improve efficiency of secondary use of EHR and consequently clinical outcome analysis. While recent studies provide specific examples that demonstrate a proof of concept that NLP techniques have tremendous benefit to capture key information from clinical text, to our knowledge there is no previous scalable evidence showing a clinical utility assessment of these studies. Not only do we compare results with the “gold standard” of manual annotations in the typical test environment for assessing model development, we also uniquely report results from a real-world clinical application to impute missing stage information in our cancer registry records. The pipeline improved missing stage information in the CDW from 32% missing to only 22% (recovering 21–71% of missing values). These results suggest that NLP-extracted data provides an avenue for recovering data missing in the EHR or cancer registries, generating a structured item available to the scientific research community as well as a potential input to real-time clinical decision aides and risk calculators.

Importantly, recent literature has highlighted important differences in clinical documentation by patient demographics (34). A recent study highlighted that Black patients had significantly fewer notes compared to non-Hispanic Whites. The impact of such differences can affect the reliability of NLP models across populations. Future research regarding the sentiment, frequency, and quality of notes associated with staging is needed to better understand model reliability.

This study has limitations. First, the patient cohort consists mostly of early stage cancer patients, reflective of the distribution of prostate cancer diagnoses in the US, and this could impact the classification tasks due to these class imbalances in the dataset, especially between N0 and N1. Although the staging distribution in the cohort was skewed, it is one of the largest real-world prostate cancer cohorts studied to date. Second, the NLP models were created and validated from a single institution which may limit generalizability. While it is possible that characteristics of the local patient population or clinical practice preferences play a role, the clinical terms used in the algorithms will be disseminated in a public repository (i.e., GitHub) and were vetted by multiple clinicians and urological nurses. Nevertheless, future work is needed to test the models in other healthcare systems to assess generalizability. Importantly, future directions should include benchmarking our model against* other baseline models, such as https://github.com/ClarityNLP/ClarityNLP/blob/master/docs/developer_guide/algorithms/tnm_stage_finder.rst. Finally, the methods developed show excellent performance characteristics, but are not error free, since there were some disagreements between the imputed stage and manually annotated records. However, the error rates in stage assignment by the models is comparable or better than that observed in cancer registries where recorded stages are compared with those reviewed by an expert panel (13). Despite these limitations, this work advances the knowledge of automated cancer stage extraction from clinical narratives.

## Conclusion and Future Work

Cancer stage is critical for determining prognosis and treatment options in newly diagnosed cancer patients; however, it is not routinely captured as structured data, but is often only available in free text clinical reports within the EHR. To facilitate the expanded use of these real-world data, advanced methods are needed to extract relevant data features from EHR. This study demonstrates that the automated extraction of TNM stage information using NLP and ML approaches achieved high accuracy, at levels comparable with manual chart review by clinical experts, and successfully improved the level of missing values in a cancer registry. This work provides a basis for automated extraction of cancer stage from free text reports to improve registries, thereby driving observational research, patient selection for clinical trials, or even enable bedside tools like risk calculators and clinical decision aides.

## Data Availability Statement

The datasets presented in this article are not readily available because the data used in this study contain patient identifiers and therefore are not available to the general public. Requests to access the datasets should be directed to boussard@stanford.edu.

## Ethics Statement

The study was approved by the Stanford Univerisy's Institutional Review Board. Written informed consent for participation was not required for this study in accordance with the national legislation and the institutional requirements.

## Author Contributions

TH-B and JB conceived the project. TH-B directed the project. SB, MS, and CM collected the data. SB and TH-B analyzed and evaluated the data and take responsibility for both the integrity of the data and the accuracy of the data analysis. SB drafted the paper. All authors reviewed and approved the manuscript.

## Funding

Research reported in this publication was supported by the Stanford AstraZeneca Research grant and National Cancer Institute of the National Institutes of Health under Award Number R01CA183962. The content is solely the responsibility of the authors and does not necessarily represent the official views of the National Institutes of Health and AstraZeneca.

## Conflict of Interest

The authors declare that the research was conducted in the absence of any commercial or financial relationships that could be construed as a potential conflict of interest.

## Publisher's Note

All claims expressed in this article are solely those of the authors and do not necessarily represent those of their affiliated organizations, or those of the publisher, the editors and the reviewers. Any product that may be evaluated in this article, or claim that may be made by its manufacturer, is not guaranteed or endorsed by the publisher.

## Supplementary Material

Supplementary Material 1 Study design schema.
