# Supplementary figures and images for: Expanding the Secondary Use of Prostate Cancer Real World Data: Automated Classifiers for Clinical and Pathological Stage
Source: Front Digit Health. 2022 Jun 2;4:793316. doi: 10.3389/fdgth.2022.793316 (PMC9201076; doi:10.3389/fdgth.2022.793316)

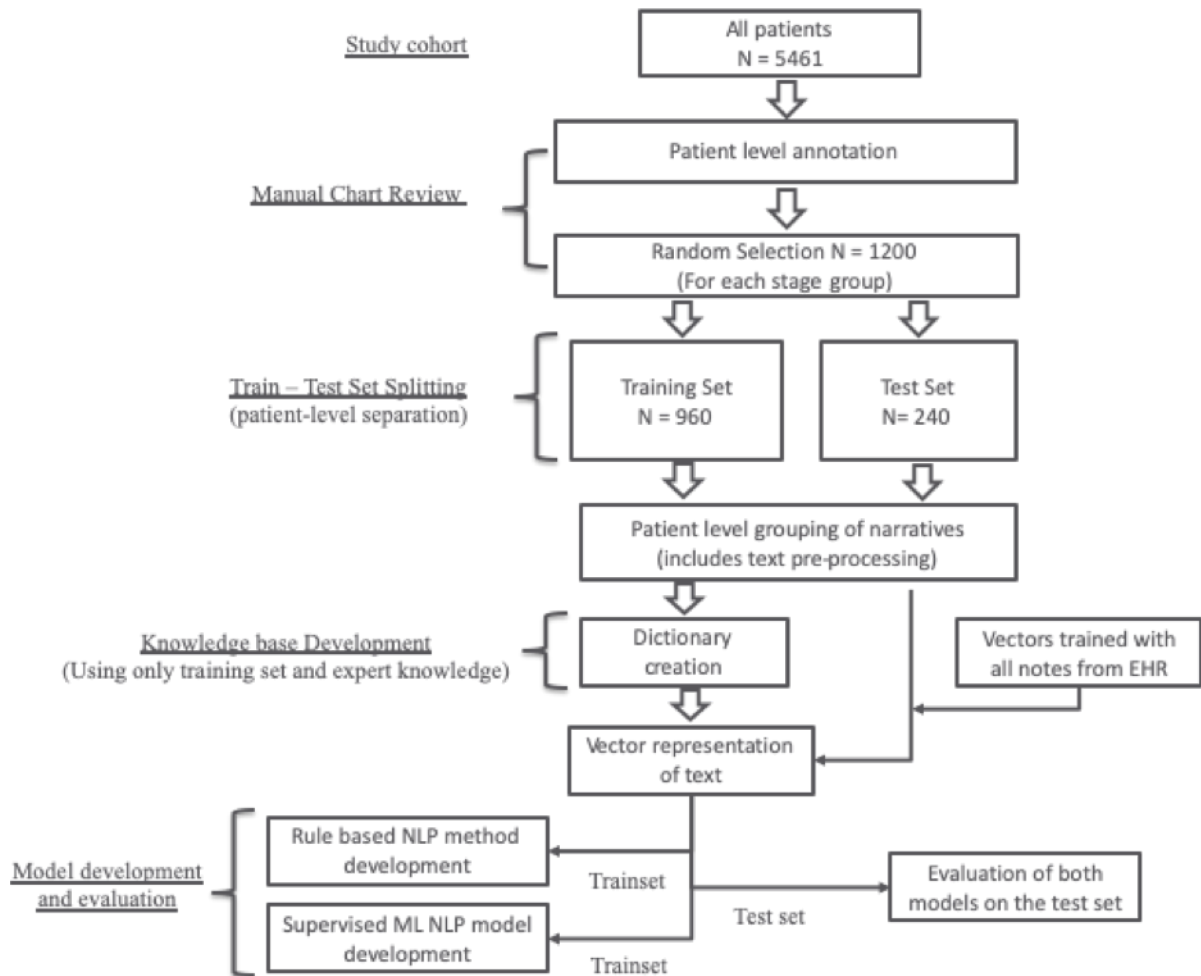

Supplement: Supplementary Material 1 — Study design schema. [file Data_Sheet_1.PDF]
